# Modified Whole Effluent Toxicity Test to Assess and Decouple Wastewater Effects from Environmental Gradients

**DOI:** 10.1371/journal.pone.0066285

**Published:** 2013-06-05

**Authors:** Sebastián Sauco, Julio Gómez, Francisco R. Barboza, Diego Lercari, Omar Defeo

**Affiliations:** UNDECIMAR, Departamento de Ecología y Evolución, Facultad de Ciencias, Universidad de la República, Montevideo, Uruguay; Texas Tech University, United States of America

## Abstract

Environmental gradients and wastewater discharges produce aggregated effects on marine populations, obscuring the detection of human impact. Classical assessment methods do not include environmental effects in toxicity tests designs, which could lead to incorrect conclusions. We proposed a modified Whole Effluent Toxicity test (mWET) that includes environmental gradients in addition to effluent dilutions, together with the application of Generalized Linear Mixed Models (GLMM) to assess and decouple those effects. We tested this approach, analyzing the lethal effects of wastewater on a marine sandy beach bivalve affected by an artificial canal freshwater discharge used for rice crops irrigation. To this end, we compared bivalve mortality between canal water dilutions (CWd) and salinity controls (SC: without canal water). CWd were prepared by diluting the water effluent (sampled during the pesticide application period) with artificial marine water. The salinity gradient was included in the design by achieving the same final salinities in both CWd and SC, allowing us to account for the effects of salinity by including this variable as a random factor in the GLMM. Our approach detected significantly higher mortalities in CWd, indicating potential toxic effects of the effluent discharge. mWET represents an improvement over the internationally standardized WET tests, since it considers environmental variability and uses appropriate statistical analyses.

## Introduction

A large proportion of human activities are dependent on the goods and services provided by coastal ecosystems, modifying their biological, physical and chemical characteristics [1], [2]. Urbanization and human enterprises like industry and agriculture alter biogeochemical cycles, destroy natural habitats and promote biodiversity depletion in coastal ecosystems, due to the release of nutrients and synthetic chemicals, or changes in water salinity, temperature and dissolved oxygen [2], [3]. The ongoing increase of human population and their adverse effects on coasts [4] demands cheap, rapid and appropriate screening approaches for rapid and timely detection of potential environmental impacts.

The detection of human impacts on the biota inhabiting aquatic ecosystems includes the problem of identifying the effects from multiple anthropogenic stressors and the challenge of distinguishing them from the consequences of environmental gradients (e.g. freshwater discharges on marine systems) [5]. Traditional approaches for assessing the impact of pollution in aquatic systems, use expensive and complex analytical methods to obtain the basic information to identify which sites and chemicals are problematic [6]. Since the 80s, whole effluent toxicity (WET) screening methods have provided a cost-effective solution to determine the effects of toxic unknown pollutants, indicating the presence/absence of effects before investing large sums of money on specific analytical techniques (not always capable of identifying all potential pollutants) [7]. However, these and other approaches currently applied are incapable of dealing with the effects of environmental gradients (e.g. salinity), that obscure pollutant effects, which can lead to incorrect conclusions [8], [9]. This calls for the reassessment of experimental designs and statistical tools used to detect anthropogenic impacts.

Our goal was to modify WET tests by recreating an experimental salinity gradient to decouple this environmental effect from those produced by a mixture of unknown pollutants, using Generalized Linear Mixed Models (GLMM). To this end, we assessed the lethal effects from wastewater on a marine sandy beach bivalve affected by an artificial canal (AC) freshwater discharge used for rice crops irrigation. The canal water collected during pesticide application periods (November and December of 2011) was used to prepare canal water dilutions (CWd) with an artificial marine solution. Salinity controls (SC), without canal water were made on a separated set of dilutions. The inclusion of the salinity gradient in the experimental design achieved by setting the same final salinities in both CWd and SC, allowed us to account for salinity effects through the use of GLMM.

## Materials and Methods

### Ethics Statement

No specific ethical or institutional permits were required to carry out the experimental studies according to Uruguayan Law N° 18.611, since invertebrate species are not protected by any legal regulation. Sampling was conducted outside protected areas and the experimental studies did not involve endangered or protected species.

### Model Species and Study Area

**Figure 1:**
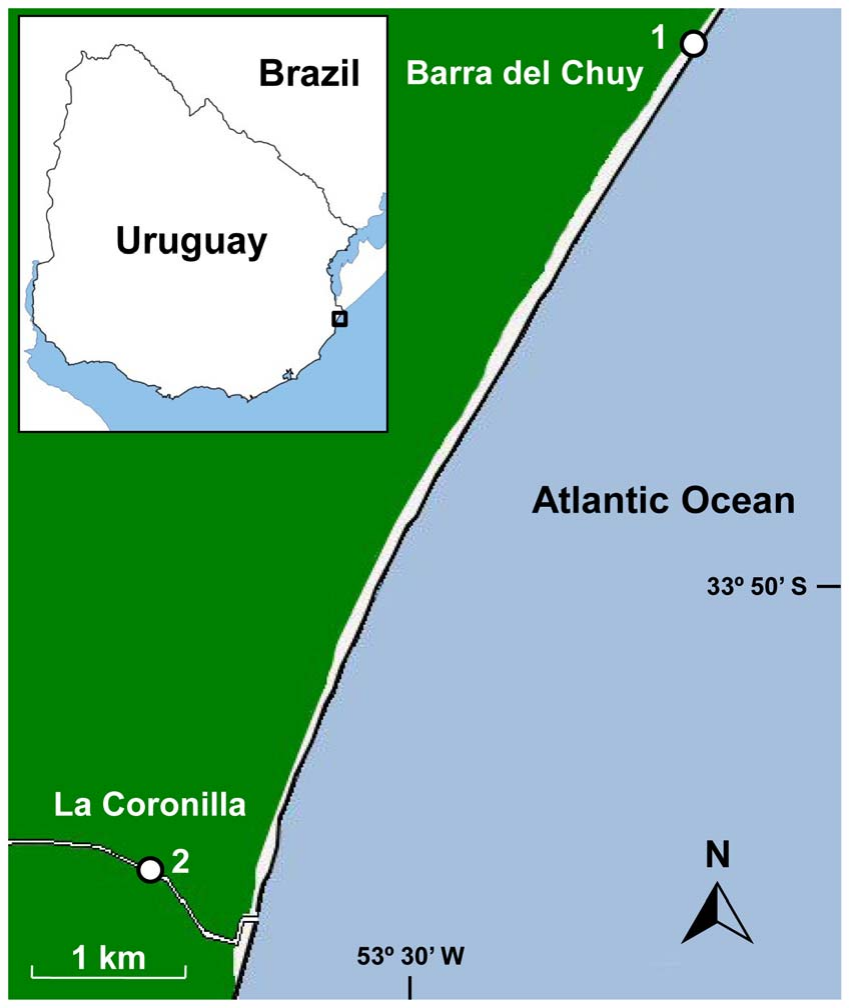
Study area. [(1)] Extraction site of bivalves in La Coronilla-Barra del Chuy sandy beach and [(2)] collection site of the effluent water in the Andreoni canal.

The sandy beach infaunal marine bivalve *Mesodesma mactroides* inhabits the intertidal zone of dissipative sandy beaches from Brazil to Argentina, being harvested throughout its distribution range [10]. In Uruguay, *M. mactroides* occurs mainly on 22 km of an uninterrupted exposed sandy beach, from Barra del Chuy (33° 40′S; 53° 20′W) to La Coronilla (33° 50′S; 53° 27′W), on the eastern Atlantic coast (Fig. 1A and 1B) [11]. This bivalve is subject to artisanal harvesting and constitutes an economic activity with significant socio-economic value [12]. The microtidal dissipative beach of La Coronilla-Barra del Chuy (LC-BC) has a gentle slope, fine well-sorted sands and a wide surf zone [13]. The man-made Andreoni canal, discharges in a SW-NE direction along LC-BC sandy beach generating a strong longshore salinity gradient (Fig. 1C) [14]. The variable flow, used for rice crops irrigation and cattle rearing, depends mainly on rainfall events and a system of independent hatches used to export water from flooded paddy fields [15]. This condition leads to a meso-scale artificial estuarine effect along the LC-BC, which impacts the macrofaunal community structure. The consequences of this man-made canal include habitat degradation, reduced diversity, abundance, survival, growth and fecundity of several macrofauna species [16]. The above evidence emphasizes the Andreoni canal freshwater inputs as the primary cause of the negative effects documented on the resident sandy beach community. Herbicide residues were detected in the canal mouth [17]. The final section of this canal (c.a. 1.5 km), towards its mouth, runs adjacent to La Coronilla town which has no drainage systems [18], [19]. These environmental pressures could act simultaneously to produce sublethal and/or lethal effects on bivalves.

### Sampling

Some 150 commercial size bivalves (50.16–65.89 mm in length) were collected in November and December 2011, at 13 km from the artificial discharge, previously defined as an undisturbed site (Fig. 1B) [20]. The organisms were put into a 100 L container filled with wet sand and transported to the laboratory in optimal conditions. In the experimental facility, they were placed in three 70 L aquariums for acclimation. Water from the canal was obtained for both periods at the same site (Fig. 1C) and transported on ice-coated containers to minimize bacterial activity.

### Acclimation Period and Purge Time Estimation

A purge time test was conducted in order to assess the time period for elimination of all the sediment stored inside the organisms. To this end, six replicates including five organisms each were placed in a 6 L container with artificial marine water (AMW) prepared at salinity values registered at the extraction site (salinity of 27 ppt) and left for 24 h. After this, bivalves were extracted and reincorporated in newly prepared AMW with the same previous salinity, for another 24 h purge period. The remaining water from each 24 h purge period was filtered through a 45 µm pore diameter filter and the retained sediment weighed to the nearest 0.001 g. The protocol was repeated until no sand was observed in the filter under optic microscope. At the end of the test, we obtained the individual sand purge weight for each replicate and time period. This time was defined as the acclimation period prior to the implementation of the definitive toxicity test, so bivalves do not need to expend metabolic energy in purging during these tests. We estimated that a minimum of six days of purging time, with a 24 h AMW renewal, is needed for commercial bivalves to remove the entire internal sediment load (Fig. S1). The sediment purge exponentially decreased with time. Based on this, seven days for laboratory acclimation was considered enough to achieve a sediment-free commercial bivalve.

### Definitive Screening Toxicity Tests: Protocol, Design and Data Analysis

Range finding tests were done in order to establish salinity concentrations for the definitive screening toxicity tests. Seven days of acclimation at salinity of 27 ppt and 17°C was performed before each toxicity test. Water was renewed every two days and salinity, temperature, dissolved oxygen and pH were registered during this period. Photoperiod was 12∶12 h light-dark cycle.

**Figure 2:**
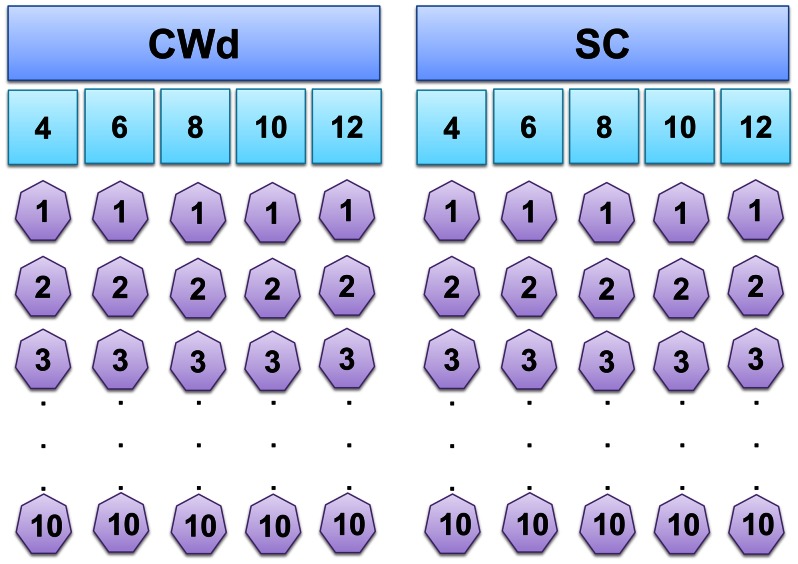
Modified Whole Effluent Toxicity design. Canal water (CWd) and salinity controls dilutions (SC: without canal water) were the fixed terms in the GLMM. The inclusion of a salinity gradient (4, 6, 8, 10, and 12 ppt) on both dilutions allowed accounting for salinity effects, setting this variable as a random factor. Ten replicates (one individual in each one) per salinity level were included.

Two modified WET (mWET) toxicity tests were performed to decouple the lethal effects of freshwater from wastewater. mWET tests were carried out in November and December of 2011, during the rice crop growing season when pesticides were detected in the canal water [17]. We prepared five SC and CWd achieving the same final salinity (4, 6, 8, 10 and 12 ppt), thus including the salinity gradient in both dilutions (Fig. 2). SC and CWd were made by diluting an artificial marine solution (salinity of 27 ppt) with deionized water and water obtained from the AC, respectively. To achieve the same final salinity on both dilutions (i.e. SC and CWd) we added the same volume of each diluent to the AMW solution (Table S1). Thus, the canal discharge gradient was simulated by the CWd and considered as a black box in terms of pollutant composition and their influence on the whole effluent toxicity. Ten replicates comprised by one individual each (50.16–65.89 mm in length) were randomly assigned for each salinity concentration in both SC and CWd. Negative controls for SC (10 replicates) were prepared with artificial marine water at salinity of 27 ppt, which could not admit more than 10% of mortality at the end of the toxicity test [21]. Operational definitions for bivalve mortality recognition were established as follows: 1) if valves were closed, the clam was alive; 2) If valves were open and siphon or foot movements were observed then the clam was alive; 3) If valves were open and movements were not observed, the clam was considered alive if it responded to an external stimulation (a soft touch on foot or siphons); and 4) if clams did not respond to external stimuli then the clam was considered dead (Fig. S2). As in the acclimation period, salinity, temperature, dissolved oxygen and pH were controlled in all replicates during mWET toxicity tests (Table S2). Adequate quality assurance/quality control (QA/QC) practices were followed in all aspects of the study, from field sampling to laboratory routine activities and data entry, following USEPA general guidance on good laboratory practices related to toxicity testing [21].

Experimental data were analyzed through a GLMM using the lme4 R package [22]. A binomial distribution and its canonical link function (logit) were implemented to model individual bivalve mortality, which allowed us to express the results in terms of odd ratios as an approximation of the relative risk. The presence/absence of canal wastewater was set as the main factor and included in the fixed terms. Freshwater effects were decoupled from those produced by canal wastewater by including salinity as a random factor. Interactive effects between wastewater and salinity were initially tested, in order to determine its inclusion in the model. Temporal variability of the response variable was considered by including the experimental time as random factor.

## Results

GLMM showed a significant increase in bivalve mortality in November, meaning that the hazard due to canal wastewater exposure was significantly higher than the isolated effects generated by low salinities (Table 1). 100% mortality was observed in CWd at salinities of 4 and 6 ppt, whereas only 10% died at salinity of 8 ppt (Table S3). In the SC, 100% mortality was only registered at salinity of 4 ppt. Mortality did not differ between CWd and the SC for December, even though there was a two-fold extra hazard due to CWd exposure (Table 1). Mortality due to CWd was 80% and 40% at salinities of 4 and 6 ppt respectively, whereas 60% mortality was registered in the SC at salinity of 4 ppt (Table S3). Mortality was not registered for salinities ranging from 6 to 12 ppt in the SC.

**Table 1:** Generalized Linear Mixed Models results for experimental bivalve mortality.

Fixed effects	Estimate	Std. Error	z value	Odd ratio
(Intercept)	−23.4**	7.9	−2.9	141.0
CWd _N_	4.9***	1.2	4.2	
(Intercept)	−6.6***	1.7	−3.8	2.5
CWd _D_	0.9	0.6	1.6	

**<0.01; *** <0.001

CWd _N_, CWd _D_: Canal water dilutions for November and December of 2011.

## Discussion

We provide a modification of the whole effluent toxicity test (GLMM and salinity gradient), which allows to decouple wastewater effects from natural environmental gradients. A mWET toxicity test and the use of appropriate statistical analyses let us separate the lethal effects of a mixture of unknown stressors from those produced by a freshwater discharge, on a marine sandy beach bivalve. The relevance of our research relies on: i) the development of a rapid and low-cost screening approach based on acute toxicity tests and the application of GLMM to assess adverse biological effects of wastewaters adjusting natural sources of variability that inevitably act in field; and ii) the improvement of early-detection techniques of pollution, extremely necessary to allow correct decision making processes, in order to mitigate the increasing anthropogenic impact that threatens coastal ecosystems.

### Specific Results

Higher CWd mortality in the November assessment could arise from a set of undetermined lethal causes acting simultaneously. However, some sources of environmental stressors from anthropogenic activities detected in the canal watershed can be pointed out as potential lethal hazards. Extensive agriculture practices could potentially be a source of herbicide residues [17] and trace metals from fertilizers [23]. Those stressors in the aquatic environment could arise from polluted soils via run off processes [24]. The lack of differences in bivalve mortality between treatments for the December toxicity test suggests equally lethal effects from CWd and freshwater. Even though our study considered two specific moments during the period of pesticide application in the field, in order to determine the capacity of the mWET test to detect potential toxic effects, additional toxicity tests are required to consistently determine the existence of biological adverse effects [9].

### Advantages and Perspectives of The Method

The creation of an environmental gradient (here a salinity gradient) in a WET experimental design (see Fig. 1), allowed us to adjust salinity effects through an appropriate statistical method. The application of a GLMM has 3 main advantages: i) it considers a Binomial distribution that characterizes individual replicated experiments, and was applied here to avoid the water impairment in pooled organisms designs, ii) it expresses the results obtained in terms of odd ratios; and iii) removes the effects of an environmental variable, adjusting the variability generated by this factor, without consuming a great amount of degrees of freedom, improving the statistical power of the method [25].

The flexibility of this mWET provides the possibility to implement it on different biological indicators and measuring lethal or sub-lethal responses. Even though our work was focused on lethal effects of wastewater, sublethal responses could be considered, since they can cause severe alterations in organisms' traits and in consequence on a biological community, without producing changes in species abundance [26]. The ability of the proposed method to incorporate a wide range of environmental sources of variability is an important advantage. In this sense, temperature, salinity (in our case) and other variables that generate gradients, can be recreated, being only necessary to conduct preliminary experiments to define the range of action on the selected biological indicator. Following the same idea, and depending on the scientific question, it is possible to include more than one gradient into the same mWET design.

The evaluation of toxic effects under different environmental conditions on aquatic ecosystems requires new integrative approaches. Existing methods (e.g. classical WET tests) assess toxic levels of pollutants without considering environmental gradients frequently present in natural ecosystems. Hence, existing experimental designs are incapable of recreating crucial details observed in field, obtaining weak conclusions about the impact suffered by species or systems of interest. Recently, several authors have begun to recognize the potential interaction between environmental factors and pollutants and how abiotic variables like salinity are capable of modifying the activity of pesticides and the bioavailability of trace metals [26], [27], [28], [29], [30], [31]. However, no efforts have been made to incorporate this aspect in internationally standardized toxicity tests. In addition, recommended manuals to analyze potential toxic effects, suggest statistical tools derived from the general linear model, that require complex transformations in order to cope with the non-Gaussian distribution that characterizes the major part of biological responses. In this context, our mWET test represents an improvement that classical assessments require in order to guarantee the accurate identification of human impact on coastal and other ecosystems.

## Supporting Information

Figure S1 **Purge time.** Relationship between the internal sediment burden and purging time for the sandy beach bivalve (y =  a·x^b^, R[2]  = 0.82). Six days takes an individual to purge the entire sediment burden with 24 h artificial marine water renewal.
(TIF)

Figure S2 **Operational definitions.** (A) Closed alive bivalve; (B) open alive bivalve; and (C) open dead bivalve. The latter state is recognizable from degradation tissue and mantle border damage.
(TIF)

Table S1 **Salinity gradient included in canal water dilutions (CWd) and salinity controls (SC).**
(DOC)

Table S2 **Range values (minimum-maximum) for the variables measured in each salinity level (4, 6, 8, 10 and 12 ppt) during November and December mWET tests.** SC: salinity controls; CWd: canal water dilutions.
(DOC)

Table S3 **Bivalve mortalities (%) registered in salinity controls (SC) and canal water dilutions (CWd) for November and December of 2011.**
(DOC)
