# Supplementary figures and images for: Modified Whole Effluent Toxicity Test to Assess and Decouple Wastewater Effects from Environmental Gradients
Source: PLoS One. 2013 Jun 5;8(6):e66285. doi: 10.1371/journal.pone.0066285 (PMC3673937; doi:10.1371/journal.pone.0066285)

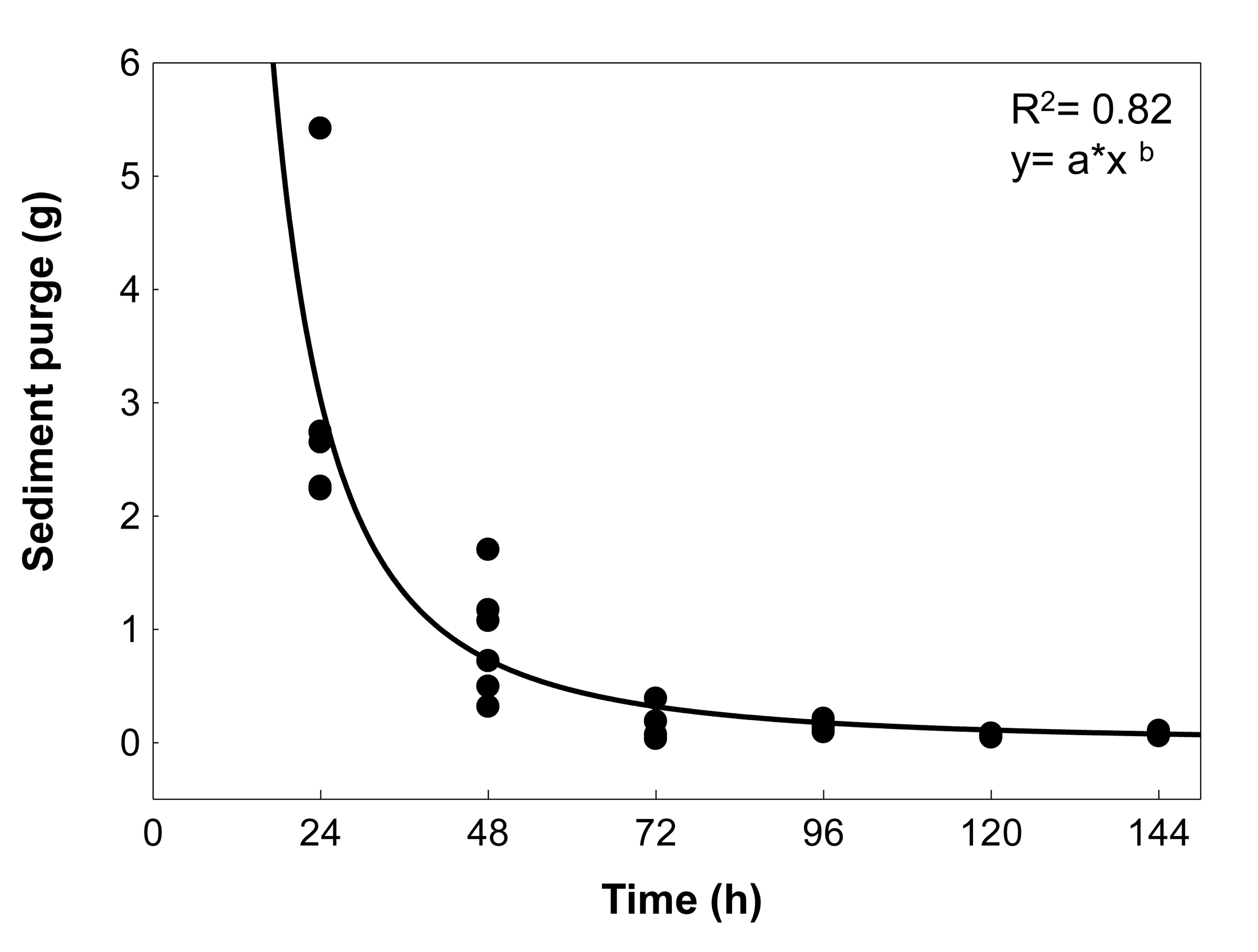

Supplement: Figure S1 — Purge time. Relationship between the internal sediment burden and purging time for the sandy beach bivalve (y = a·xb, R[2] = 0.82). Six days takes an individual to purge the entire sediment burden with 24 h artificial marine water renewal. (TIF) [file pone.0066285.s001.tif]

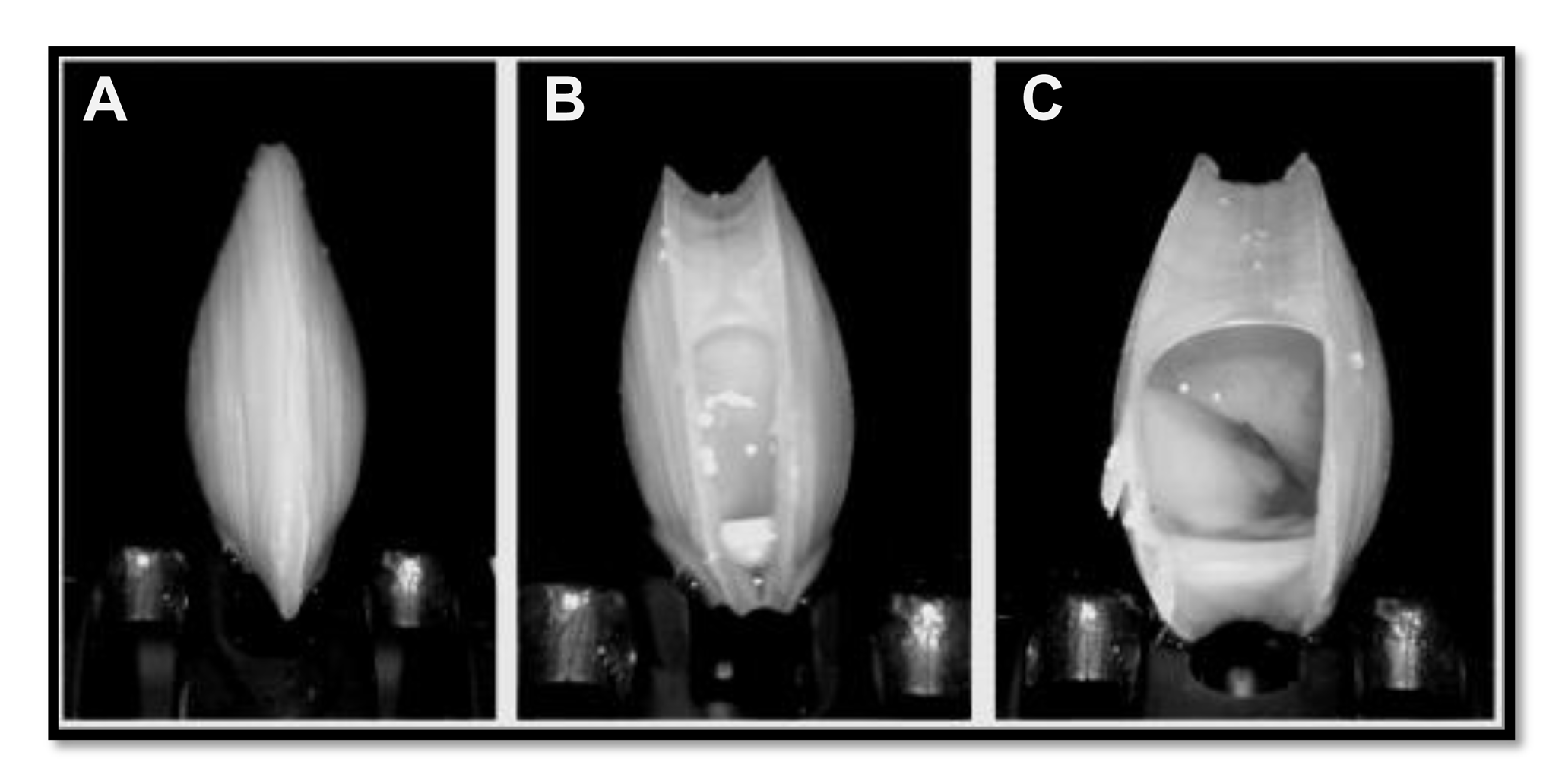

Supplement: Figure S2 — Operational definitions. (A) Closed alive bivalve; (B) open alive bivalve; and (C) open dead bivalve. The latter state is recognizable from degradation tissue and mantle border damage. (TIF) [file pone.0066285.s002.tif]
