# Supplementary material for: Modified Whole Effluent Toxicity Test to Assess and Decouple Wastewater Effects from Environmental Gradients
Source: PLoS One. 2013 Jun 5;8(6):e66285. doi: 10.1371/journal.pone.0066285 (PMC3673937; doi:10.1371/journal.pone.0066285)
Supplement: Table S1 — Salinity gradient included in canal water dilutions (CWd) and salinity controls (SC). (DOC) [file pone.0066285.s003.doc]

Table S1. Salinity gradient included in canal water dilutions (CWd) and salinity controls (SC).

| Salinity Gradient | 12 | 10 | 8 | 6 | 4 |
| --- | --- | --- | --- | --- | --- |
| CWd (%) | 55 | 63 | 70 | 78 | 85 |
| SC (%) | 55 | 63 | 70 | 78 | 85 |
