# Supplementary material for: Modified Whole Effluent Toxicity Test to Assess and Decouple Wastewater Effects from Environmental Gradients
Source: PLoS One. 2013 Jun 5;8(6):e66285. doi: 10.1371/journal.pone.0066285 (PMC3673937; doi:10.1371/journal.pone.0066285)
Supplement: Table S2 — Range values (minimum-maximum) for the variables measured in each salinity level (4, 6, 8, 10 and 12 ppt) during November and December mWET tests. SC: salinity controls; CWd: canal water dilutions. (DOC) [file pone.0066285.s004.doc]

**Table S2. Range values (minimum - maximum) for the variables measured in each salinity level (4, 6, 8, 10 and 12) during November and December mWET tests. SC: salinity controls; CWd: canal water dilutions.**

| **November** | **CWd** | | | | | **SC** | | | | |
| --- | --- | --- | --- | --- | --- | --- | --- | --- | --- | --- |
|  | **4** | **6** | **8** | **10** | **12** | **4** | **6** | **8** | **10** | **12** |
| **Salinity** | 4.0 - 4.2 | 6.0 - 6.2 | 8.0 - 8.2 | 10.0 - 10.2 | 12.0 - 12.2 | 4.0 - 4.2 | 6.0 - 6.2 | 8.0 - 8.2 | 10.0 - 10.3 | 12.0 - 12.3 |
| **Temperature (°C)** | 16.8 - 17.3 | 16.9 - 17.4 | 16.8 - 17.5 | 16.9 - 17.4 | 16.9 - 17.5 | 16.7 - 17.5 | 16.8 - 17.5 | 16.7 - 17.5 | 16.7 - 17.5 | 16.8 - 17.4 |
| **Dissolved oxygen (mg/L)** | 7.56 - 8.55 | 6.87 - 8.24 | 8.23 - 8.71 | 8.11 - 9.10 | 7.52 - 9.24 | 6.95 - 8.35 | 7.74 - 8.42 | 7.87 - 8.28 | 7.83 - 8.33 | 7.90 - 8.38 |
| **pH** | 7.84 - 8.28 | 7.69 - 8.47 | 7.74 - 8.30 | 7.70 - 8.31 | 7.70 - 8.35 | 7.65 - 8.14 | 7.86 - 8.15 | 7.74 - 8.24 | 7.65 - 8.18 | 7.63 - 8.14 |
|  |  | | | | | | | | | |
| **December** | **CWd** | | | | | **SC** | | | | |
|  | **4** | **6** | **8** | **10** | **12** | **4** | **6** | **8** | **10** | **12** |
| **Salinity** | 4.0 - 4.2 | 6.0 - 6.2 | 8.0 -8.2 | 10.0 - 10.2 | 12.0 - 12.2 | 4.0 - 4.2 | 6.0 - 6.2 | 8.0 -8.2 | 10 - 10.2 | 12.0 - 12.2 |
| **Temperature (°C)** | 16.9 - 17.4 | 16.9 - 17.4 | 16.9 - 17.5 | 16.9 - 17.5 | 16.9 - 17.4 | 17.1 - 17.4 | 17.2 - 17.4 | 16.9 - 17.4 | 17.1- 17.4 | 17.2 - 17.4 |
| **Dissolved oxygen (mg/L)** | 7.74 - 8.60 | 7.84 - 8.44 | 7.20 - 8.80 | 7.76 - 8.10 | 7.83 - 8.42 | 7.74 - 8.60 | 7.63 - 8.49 | 8.00 - 8.20 | 8.14 - 8.74 | 7.61 - 8.62 |
| **pH** | 7.54 - 8.18 | 7.72 - 8.27 | 7.63 - 8.24 | 7.59 - 8.23 | 7.74 - 8.24 | 7.51 - 8.14 | 7.58 - 8.20 | 7.85 - 8.21 | 7.63 - 8.19 | 7.74 - 8.17 |
