# Supplementary material for: Modified Whole Effluent Toxicity Test to Assess and Decouple Wastewater Effects from Environmental Gradients
Source: PLoS One. 2013 Jun 5;8(6):e66285. doi: 10.1371/journal.pone.0066285 (PMC3673937; doi:10.1371/journal.pone.0066285)
Supplement: Table S3 — Bivalve mortalities (%) registered in salinity controls (SC) and canal water dilutions (CWd) for November and December of 2011. (DOC) [file pone.0066285.s005.doc]

**Table S3. Bivalve mortalities (%) registered in salinity controls (SC) and canal water dilutions (CWd) for November and December of 2011.**

| Salinity Gradient |  | 12 | 10 | 8 | 6 | 4 |
| --- | --- | --- | --- | --- | --- | --- |
| November | CWd | 0 | 0 | 10 | 100 | 100 |
|  | SC | 0 | 0 | 0 | 0 | 100 |
| December | CWd | 0 | 0 | 0 | 40 | 80 |
|  | SC | 0 | 0 | 0 | 0 | 60 |
